# The Role of Douglasectomy Instead of Random Biopsies in the Surgical Treatment of Presumed FIGO Stage I Ovarian Cancer

**DOI:** 10.3390/cancers17030419

**Published:** 2025-01-27

**Authors:** Dimitrios Tsolakidis, Dimitrios Zouzoulas, Panagiotis Tzitzis, Iliana Sofianou, Vasileios Theodoulidis, Kimon Chatzistamatiou, Tilemachos Karalis, Maria Topalidou, Eleni Timotheadou, Grigoris Grimbizis

**Affiliations:** 1 1st Department of Obstetrics & Gynecology, Aristotle University of Thessaloniki, “Papageorgiou” Hospital, 56429 Thessaloniki, Greece; 2 Radiotherapy Department, “Papageorgiou” Hospital, 56429 Thessaloniki, Greece; 3 Department of Oncology, Aristotle University of Thessaloniki, “Papageorgiou” Hospital, 56429 Thessaloniki, Greece

**Keywords:** douglasectomy, ovarian cancer, presumed FIGO stage I, survival

## Abstract

**Background/Objectives:** Douglasectomy is defined as the removal of the pelvic peritoneum of the entire pouch of Douglas. In presumed FIGO stage I ovarian cancer, isolated microscopic cancer cells might disseminate from the ovaries to their neighboring pelvic peritoneum. However, a simple hysterectomy with bilateral salpingo-oophorectomy and a staging procedure is the standard of care. This study aims to investigate the safety and feasibility of douglasectomy compared to random pelvic biopsies, and it is based on the survival of patients with early ovarian cancer. **Methods:** We retrospectively analyzed the records of patients with presumed 2018 FIGO stage I ovarian cancer who underwent surgery in the 1st Department of Obstetrics and Gynecology Clinic from 2012 to 2022. Patient characteristics and oncological and follow-up information were collected. **Results:** A total of 88 patients were categorized into two groups, namely Group A (27 patients) with douglasectomy and Group B (61 patients) with random biopsies. There was no statistically significant difference in age, BMI, comorbidities, FIGO stage, intraoperative blood loss, and ICU admittance between the two groups. Conversely, patients with en bloc hysterectomy–douglasectomy had statistically significant higher pre-operative CA-125 values, surgery duration, rate of postoperative complications, and hospital stay. Concerning survival rates, there was a statistically significant difference in disease-free survival (*p* = 0.033), but no difference was observed in overall survival (*p* = 0.66). **Conclusions:** En bloc removal of the pelvic peritoneum of the entire pouch of Douglas with the uterus is a safe and feasible technique during surgery for early ovarian cancer, which leads to improved disease-free survival and local control.

## Simple Summary

En bloc removal of the whole peritoneum of the pouch of Douglas with the uterus instead of blind random biopsies is proposed for the treatment of early ovarian cancer patients. The rationale for this out-of-the-box procedure is based on the “seed and soil” hypothesis of the development of peritoneal carcinomatosis. Epithelial ovarian cancer cells from the primary tumor can exfoliate in the pelvic peritoneum and then disseminate in the peritoneal cavity through the clockwise peritoneal fluid circulation. The aim of this study is to investigate the safety and feasibility of douglasectomy and its impact on survival rates in presumed 2018 FIGO stage I ovarian cancer patients.

## 1. Introduction

The risk of developing ovarian cancer is low, at approximately 1%. Nevertheless, only 20% of the patients are diagnosed at an early stage [1]. The prognosis of early-stage disease is significantly better from advanced stages, with a 5-year survival rate of around 90% [2]. The importance of comprehensive staging in early ovarian cancer was described by Young et al. in 1983 [3] and was confirmed by later studies [4,5,6], where upstaging of the disease ranged from 30% to 50% of the cases. The high prognostic value of a complete staging procedure on patients’ outcomes has been verified by several studies [7,8,9]. Furthermore, data about recurrence in early-stage ovarian cancer revealed that 25–28% of these patients will experience a relapse [10]. Concerning the site of first recurrence, the majority of them will occur at the peritoneum, suggesting that more efforts should be directed towards an extensive and radical surgery at the peritoneum [11]. However, the recommendation for peritoneal staging, when no macroscopic disease is present, is to perform blind biopsies of the vesicouterine pouch, the pouch of Douglas, the paracolic gutters, and the hemidiaphragms [12,13].

Douglasectomy is defined as the removal of the entire peritoneum of the pouch of Douglas. This procedure is not routinely performed in gynecologic surgery, since it requires the recognition and dissection of vital structures in the pelvis, such as ureters, the hypogastric plexus, and the rectosigmoid. Intraoperative injury of the abovementioned structures can lead to increased morbidity and mortality. Douglasectomy is often used for benign conditions in gynecology, such as deeply infiltrative endometriosis of the rectovaginal septum [14] or painful uterine retroversion [15], with excellent results concerning safety and quality of life. Moreover, the en bloc removal of the whole pelvic peritoneum, including the pouch of Douglas, in order to achieve complete removal of the tumor burden in cases of locally advanced ovarian cancer, was firstly described by Hudson in 1968 [16].

On the other hand, there are no reports in the literature on the role of douglasectomy in presumed FIGO stage I ovarian cancer, thus leaving simple hysterectomy with bilateral salpingo-oophorectomy as the standard of care in the pelvis. The question arises about the implementation of douglasectomy instead of random blind biopsies from the normal appearing pelvis. Ovarian cancer may originate not only from the ovaries, which was traditionally thought to be the case, but also from dysplastic lesions in the distal parts of the fallopian tubes [17]. This proposes a dualistic model for ovarian carcinogenesis [18]. The rationale behind this is that isolated microscopic cancer cells from the fallopian tube might disseminate and implant to their neighboring pelvic peritoneum. So, the en bloc removal of the peritoneum of the pouch of Douglas with the uterus is the goal. This type of procedure was analyzed step by step [19] for the treatment of locally advanced disease, using bilateral retroperitoneal exposure, development of the paravesical and pararectal spaces, uterolysis, completion of the typical steps of a simple hysterectomy with bilateral salpingo-oophorectomy, round colpotomy from anterior to posterior, and retrograde dissection of the rectovaginal septum until the complete removal of the peritoneum of the pouch of Douglas. The aim of this study is to investigate the safety and feasibility of douglasectomy and its impact on the survival rates of patients with early ovarian cancer.

## 2. Materials and Methods

### 2.1. Study Characteristics

A retrospective analysis of women with newly diagnosed early ovarian cancer who underwent surgery in the 1st Department of Obstetrics and Gynecology, AUTh, “Papageorgiou” General Hospital, from 1 January 2012 until 31 December 2022, was performed. After careful review of the operative and the final pathology report, patients with disease confined to the ovaries with or without positive retroperitoneal lymph nodes were included in the study. So, the population consists of patients with presumed 2018 FIGO stage I ovarian cancer because of the inclusion of those that were finally upstaged to 2018 FIGO stage IIIA_1_ due to microscopic lymph node involvement. In these patients, the disease was macroscopically confined to the ovaries with no visible bulky retroperitoneal lymphadenopathy, but the final pathology report revealed positive retroperitoneal lymph nodes.

The definitive diagnosis of malignancy was established in the final pathology report, while the suspicion of ovarian cancer was set by imaging (all patients underwent an MRI pelvic scan and CT thorax–abdomen and vaginal ultrasound with IOTA score) and was confirmed by the intraoperative frozen section biopsy of the adnexal mass. All patients underwent a one-step procedure with no re-operations. The total number of patients retrieved in this period was 110. A written approval was received from the Institutional Review Board.

### 2.2. Patients

Inclusion criteria comprised the following:

- Histological confirmation of epithelial ovarian cancer.
- 2018 FIGO stage I or IIIA_1_.
- Surgical treatment at our Gynecological–Oncology Unit.

Exclusion criteria included the following:

- Prior surgery in the pelvis.
- Recurrent ovarian cancer.
- Missing important registry data.

**Figure 1:**
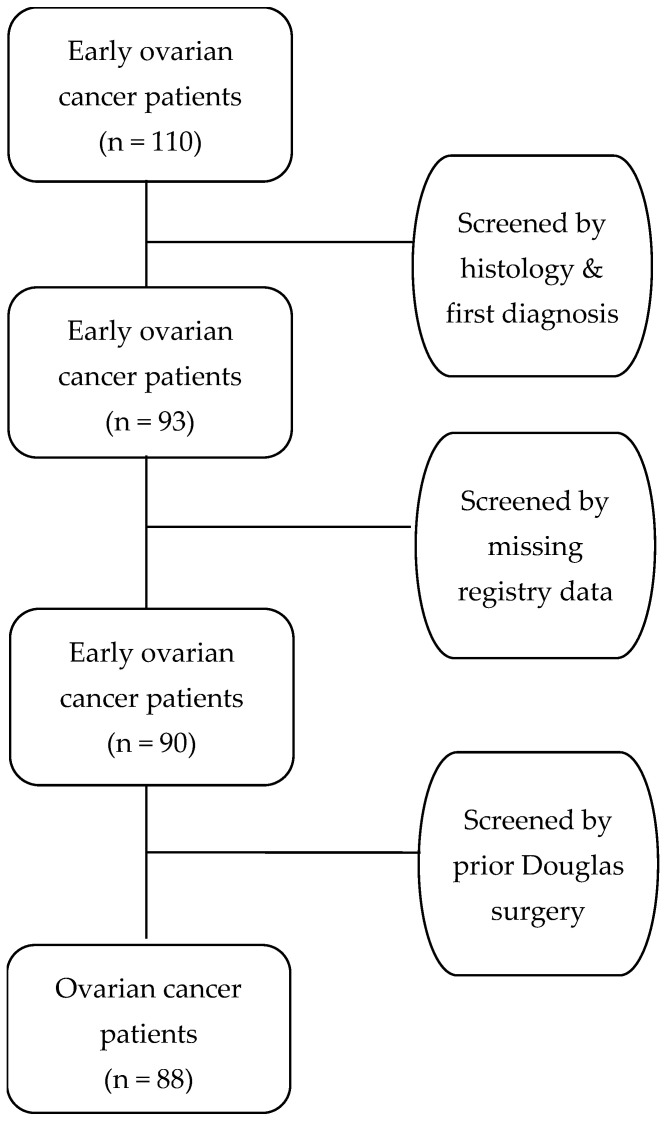
Patient selection flowchart.

As a result of the abovementioned criteria, 17 out of the 110 women with early ovarian cancer were excluded due to non-epithelial histopathological diagnosis or as a recurrence of early ovarian cancer. Moreover, 3 women were excluded because they were missing important registry data and 2 due to prior surgery in the pouch of Douglas. Hence, 88 women with presumed FIGO stage I ovarian cancer were identified as eligible for further analysis, with no duplicated data and important missing values. All patients underwent laparotomy and were divided into 2 groups; Group A underwent en bloc hysterectomy with bilateral salpingo-oophorectomy and douglasectomy and Group B (control) underwent simple hysterectomy with bilateral salpingo-oophorectomy and random pelvic biopsies. Patients in both groups also underwent staging surgery with infracolic omentectomy, pelvic and para-aortic lymphadenectomy, and peritoneal biopsies from the vesicouterine pouch, the paracolic gutters, and the hemidiaphragms. The majority of the patients in Group A were from 2018 to 2022, where douglasectomy was performed by the leading surgeon (D.T.) on all early ovarian cancer patients. All patients received adjuvant platinum-based chemotherapy, according to the international guidelines [20], except for one woman in Group B with FIGO stage IA endometrioid grade 1 ovarian cancer. The patient selection process (flowchart) is visually represented in Figure 1, providing a transparent overview of the cohort and the exclusion criteria applied.

The landmarks and surgical steps that are followed during the removal of the peritoneum of the pouch of Douglas en bloc with the uterus are described in detail below. The surgical procedure of douglasectomy starts with retroperitoneal exposure through dividing the round ligament of the uterus and the development of pararectal, paravesical, and presacral space. After ureterolysis from the posterior leaf of broad ligament, the uterine vessels are divided from their origin from the anterior branch of the interior iliac artery. Then, the prevesical peritoneum was dissected from the bladder, followed by colpectomy and retrograde rectovaginal septum dissection and rectal shaving.

### 2.3. Data Collection

Patients’ relevant information was mined over a timeline of thirty days. Our Gynecological–Oncology Unit has an online system where all the medical records are stored and are easily accessed for analysis. In order to avoid mistakes during the harvesting of data from different people over multiple days, a unique data collection sheet (Excel file) was used. The data spreadsheet included the following variables:

- Patient identification:
  o First and last name;
  o Hospital identification number.
- Age;
- Body mass index (BMI);
- Comorbidities measured by the Charlson Comorbidity Index (CCI);
- 2018 FIGO staging system classification;
- Cancer antigen 125 (CA-125) preoperative values;
- Histopathological report;
- Largest dimension of the ovarian tumor;
- Blood loss during surgery;
- Surgery duration;
- Type of surgery: douglasectomy or random peritoneal biopsies;
- Intensive care unit (ICU) admission;
- Clavien–Dindo classification for post-operative complications;
- Hospitalization;
- Time-related data:
  o Date of surgery;
  o Date of recurrence;
  o Date of last follow-up or death.

### 2.4. Statistical Analysis

All statistical analyses were performed with R statistical software (R Project for Statistical Computing) version 4.3.0. For descriptive statistics of qualitative variables, the frequency distribution procedure was used with calculation of the number of cases and percentages. Tests of normality were conducted using Shapiro–Wilk or Kolmogorov–Smirnov tests depending on sample size. On the other hand, for descriptive statistics of quantitative variables, the mean, median, interquartile range (IQR), and standard deviation were used to describe central tendency and dispersion. Disease-free (DFS) and overall survival (OS) analyses were performed using the Kaplan–Meier curves and groups were compared using the log-rank test and Cox regression. Disease-free survival was defined as the time interval between the date of surgery and date of first recurrence, while overall survival was the time interval from the date of surgery to the date of death or last follow-up. All reported *p*-values were two-tailed at a 5% significance level.

## 3. Results

This retrospective cohort study included 110 women with early ovarian cancer that were treated in the Gynecological–Oncology Unit, 1st Department of Obstetrics and Gynecology, Aristotle University of Thessaloniki, “Papageorgiou” General Hospital. After a screening of the patients based on the inclusion and exclusion criteria, 88 patients were eligible for further analysis in this study.

The population of the study was divided into two groups based on the removal of the peritoneum of the pouch of Douglas. So, Group A included 27 (30.7%) patients that underwent douglasectomy and Group B included 61 (69.3%) patients with random pelvic biopsies. Patients’ characteristics of the two groups are outlined in Table 1. The two groups were of similar age, BMI, and comorbidities, which were measured with the Charlson Comorbidity Index. Our patients were approximately 55 years old, with a median BMI of 28 (overweighted) and with mild comorbidities. Moreover, no difference was observed in terms of the histopathological subtypes, with the majority of the patients suffering from high-grade serous neoplasms. Concerning ovarian tumor size, the dimensions were extracted from the final histopathological report. The largest dimension of the tumor was captured and when both ovaries were involved, the size of the larger one was considered. No significant difference was observed between the two groups; however, patients in Group A had larger tumors. Also, there was no significant difference in the intraoperative blood loss (300cc) and the need for ICU admission after surgery. On the other hand, patients that underwent en bloc hysterectomy with bilateral salpingo-oophorectomy and douglasectomy had a significantly higher pre-operative CA-125 (Group A: 192.5 vs. Group B: 48.5) (*p* = 0.018) and a significantly longer surgery duration (Group A: 270 min vs. Group B: 180 min) (*p* < 0.01) and hospital stay (Group A: 8 days vs. Group B: 7 days) (*p* < 0.01). Furthermore, patients that underwent douglasectomy had a significantly higher rate of postoperative complications, which was measured with Clavien–Dindo classification (Group A: 17.3 vs. Group B: 15) (*p* = 0.033), but with no grade 3 complications. Specifically, in Group A, grade 2 complications included two cases of incomplete paralytic ileus, five cases of postoperative infection with fever (urinary, central venous catheter, surgical wound, diarrhea), and two cases where postoperative blood transfusion was needed.

Analyzing the distribution of the 2018 FIGO staging system of the study’s population, the majority of the patients were FIGO stage IA (*n* = 74, 84.1%) and only a fraction of patients (*n* = 14, 15.9%) were upstaged to FIGO stage IIIA_1_. There was no statistically significant difference in the FIGO sub-stages between the two groups and, specifically, FIGO stage IIIA_1_ patients were evenly divided in Group A and B (seven patients each). Moreover, no significant difference was found in the 12 cases of FIGO stage IC2-3, where four patients underwent douglasectomy (Group A) and eight random peritoneal biopsies (Group B). The aforementioned data are analyzed in Table 2.

**Figure 2:**
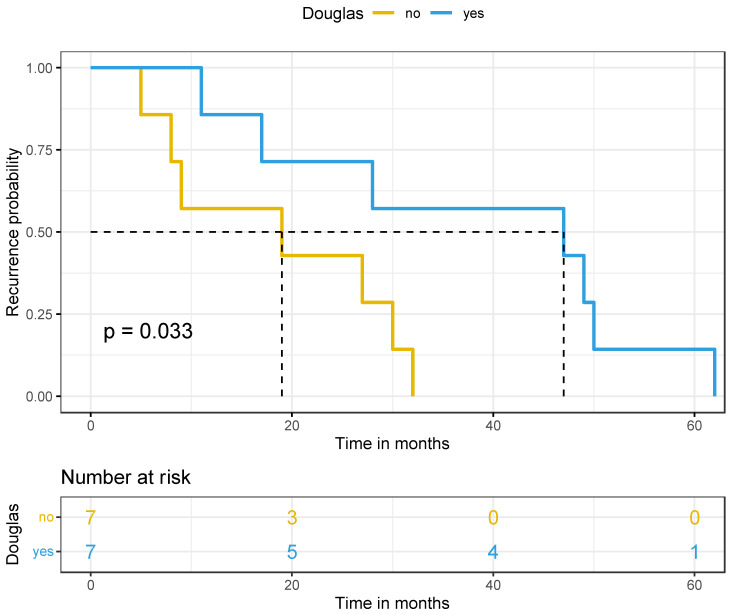
Disease-free survival (Kaplan–Meier curve).

**Figure 3:**
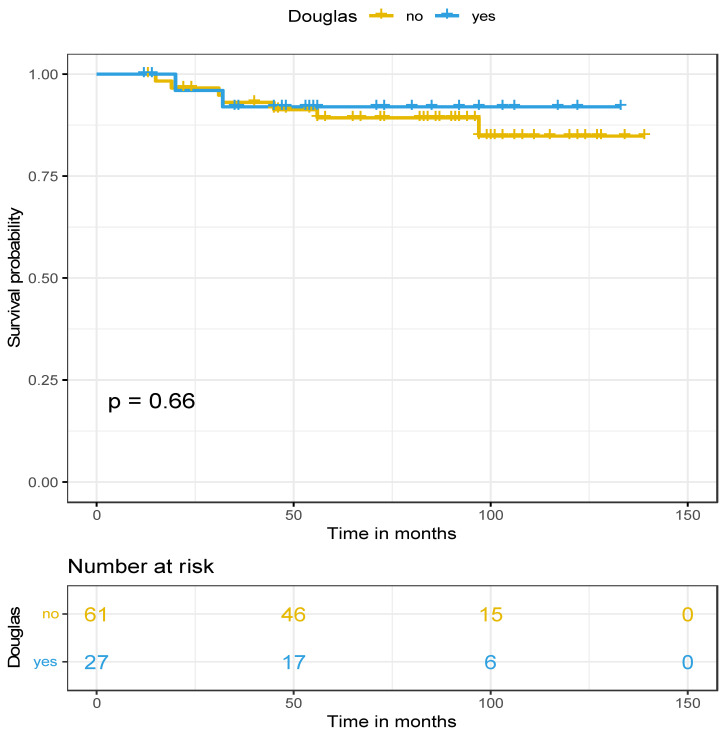
Overall survival (Kaplan–Meier curve).

The median follow-up of the cohort was 83.4 months with an IQR: 49–100.7. Regarding survival rates, there was a significant difference in disease-free survival (*p* = 0.033) in favor of the douglasectomy group, with the median DFS in Group A at 47 months and in Group B at 19 months. Concerning the first recurrence site in both groups, no relapse was found in the pelvis and especially in the pouch of Douglas in the douglasectomy group (Group A). However, four out of the seven cases of recurrence concerned retroperitoneal lymph nodes, and all patients were initially FIGO stage IIIA_1_. In contrast, in four cases out of seven in the random pelvic biopsies group (Group B), a nodule in the pelvic peritoneum was presented as the first recurrence site and only two cases of a retroperitoneal lymph node relapse, who also were initially FIGO stage IIIA_1_. Last but not least, no difference was observed in the overall survival (*p* = 0.66) between the two groups (median OS > 141 months). Nevertheless, a trend for a better overall survival was observed in favor of the douglasectomy group, because 10-year OS was approximately 92% for Group A and 85% for Group B. Survival data are presented in Figure 2 and Figure 3 for DFS and OS, respectively.

## 4. Discussion

The primary objective of our study was binary. We investigated the safety and feasibility of douglasectomy in presumed FIGO stage I and its impact on disease-free and overall survival. The rationale for this out-of-the-box procedure is that epithelial ovarian cancer tumor cells passively exfoliate from the primary site to the adjacent peritoneal cavity, which is the pouch of Douglas. We performed a retrospective analysis of the operative and final pathology report from all consecutive patients with early ovarian cancer and selected those with FIGO stage I and IIIA_1_. Then, we divided the population of the study into two groups based on the removal of the whole peritoneum of the pouch of Douglas en bloc with the uterus or not, with Group A undergoing douglasectomy and Group B undergoing random pelvic biopsies.

Eighty-eight patients were finally included in the study. From those, only a small proportion (*n* = 15, 15.9%) was upstaged at the final pathology report to FIGO stage IIIA_1_. Group A included 27 (30.7%) and Group B 61 (69.3%) patients, respectively. Even though the population in the two groups was not balanced in terms of numbers, they had similar demographic and disease characteristics (age, BMI, comorbidities, histology, tumor size, FIGO staging), therefore minimizing possible selection bias. Furthermore, the was no difference between the two groups for the intraoperative blood loss and the need for ICU admission after surgery, showing that douglasectomy, in the hands of well-trained gynecologist–oncologists with good knowledge of the anatomical landmarks of the pelvis, is a safe and feasible procedure. On the other hand, there was a significant difference between the two groups concerning pre-operative CA-125 values, surgery duration, post-operative complications, and hospital stay. Pre-operative CA-125 values were higher in the douglasectomy group. This may be due to the fact that Group A had a higher proportion of FIGO stage IIIA1 patients and also that the ovarian tumor size was larger when compared to Group B. Patients that underwent douglasectomy had longer surgery duration by approximately 90 min and longer hospital stay by one day. This could be explained by the fact that in order to perform a douglasectomy, the careful recognition and dissection of vital structures (ureters, hypogastric plexus, rectosigmoid, etc.) is needed. The rate of post-operative complications was higher in the douglasectomy group. However, no grade 3 complication was documented, a fact that empowers the idea that douglasectomy is a safe and feasible procedure.

Investigating the impact of douglasectomy on the survival rates, a long follow-up period was crucial in order to extract safe and robust results. A significantly higher DFS in favor of the douglasectomy group was found (medina DFS in Group A: 47 months vs. in Group B: 19 months). Specifically, when looking at the first recurrence site, no relapse in the pelvis (especially the pouch of Douglas) occurred in the douglasectomy group (Group A), while four cases of peritoneal recurrence presented as nodules in the pouch of Douglas in the control group with random pelvic biopsies only (Group B), which was statistically significant. This promising data highlight the possible important role of douglasectomy in limiting peritoneal recurrences and improving local disease control. In contrast, no difference was observed in OS between the two groups, mainly because an even longer follow-up period for a significant benefit in overall survival to be observed in the douglasectomy group is needed. The reason is that early ovarian cancer has per se a good prognosis. However, a trend for better overall survival in favor of the douglasectomy group was observed after 10 years at follow-up.

To our best knowledge, this is the only study in the literature that investigates the role of douglasectomy in early ovarian cancer. Recent studies [21,22,23] focusing on the development of peritoneal carcinomatosis highlight the role of the adjacent peritoneum near the primary tumor site. The idea is based on the “seed and soil” theory that was first described in 1889 by Paget [24,25], where the “seeds” are the peeled epithelial cancer cells and the peritoneum is the “soil”. The first two steps of peritoneal carcinomatosis are the detachment of epithelial ovarian cancer cells from the primary tumor into the pelvic peritoneum and the dissemination of these cells in the peritoneal cavity with the peritoneal fluid between the visceral and parietal peritoneum. Gravity and respiratory and bowel movements create an intra-abdominal flow that transfers the peritoneal fluid in a repetitive pattern from the lower to the upper abdomen [26]. So, the removal of the whole peritoneum of the pouch of Douglas can stop this process at each initial phase, preventing future peritoneal carcinomatosis.

A retrospective study [11] investigating the patterns of recurrence in ovarian cancer included some (*n* = 13, 18%) FIGO stage I patients. The sites of first recurrence, six in total, were all in the peritoneum. This finding shows the important role of the peritoneum in tumor dissemination. Moreover, another retrospective cohort study [6], where FIGO stage IA and IIIA patients (*n* = 35) were reoperated after a median time period of approximately 2 months, had a range of almost up to 12 months. The authors found that 12 (34%) patients had metastases in the pelvic peritoneum, when in the primary surgery, no evidence of disease was described in that anatomical area. Furthermore, before the implementation of different procedures during the surgical treatment of ovarian cancer, clinicians should always balance their morbidity and efficacy. In our study, douglasectomy demonstrated higher postoperative complications but with no grade 3 ones. In the literature, many procedures have been questioned about their risks and benefits. Lymphadenectomy, which was the cornerstone of ovarian cancer treatment, especially in advanced stages, is now abandoned due to higher complication rates with no survival benefit [27]. On the other hand, debulking ovarian cancer patients with a high tumor load in the upper abdomen and especially with hepatobiliary disease is often accompanied with mild-to-severe complications. However, when complete or optimal cytoreduction is accomplished, the survival benefit overshadows the possible risks [28].

This is the first study in the literature that investigates the role of douglasectomy in the surgical treatment of early ovarian cancer. All the required parameters were collected from an online system, therefore minimizing the percentage of missing important data. It is of high importance to state that the dataset on which this study was conducted comes from an ESGO-certified center for advanced ovarian cancer surgery for over a decade, thus ensuring the high quality of the data. Nevertheless, the main limitation of our study is the low number of participants, especially in the douglasectomy group, included in the final analysis and its retrospective nature.

The results of our study could have a huge impact on everyday clinical practice, because douglasectomy seems to improve DFS in early ovarian cancer patients while being a safe and feasible procedure. Future studies should also focus on FIGO sub-stages IC2-3, where tumor cells are definitely present in the fluid at the pouch of Douglas and douglasectomy could provide even more favorable survival rates for these patients. Further prospective studies are imperative to validate these promising results before douglasectomy is established in the algorithm of early ovarian cancer staging procedures.

## 5. Conclusions

En bloc douglasectomy with the uterus in early ovarian cancer patients seems to be a safe and feasible procedure that has a huge impact on disease-free survival. The rationale about this out-of-the-box procedure can be explained by the theory of peritoneal carcinomatosis development.

## Figures and Tables

**Table 1:** Patients’ characteristics.

Characteristics		Group A *n* (%) 27 (30.7%)	Group B *n* (%) 61 (69.3%)	*p*-Value
Age (years) mean (SD)		55.7 (11.7)	52.6 (14.9)	0.344
BMI (kg/m^2^) median (IQR)		28 (24.3, 32.3)	27.8 (24.1, 30.0)	0.436
CCI median (IQR)		2 (0–3)	1 (0–3)	0.311
CA-125 (U/mL) median (IQR)		192.5 (55.7, 3009)	48.5 (15.4, 186.1)	**0.018**
Ovarian tumor size (mm) median (IQR)		120 (100, 225)	100 (57.5, 185)	0.1982
Blood loss (cc) median (IQR)		300 (250, 600)	300 (200, 500)	0.068
Surgery duration (min) median (IQR)		270 (240, 300)	180 (120, 240)	**<0.001**
Histology				0.8081
	serous	17 (63%)	42 (68.8%)	
	endometrioid	8 (29.6%)	15 (24.6%)	
	other	2 (7.4%)	4 (6.6%)	
Clavien–Dindo classification median (IQR)		17.3 (12.2, 33.2)	15.0 (0, 23.4)	**0.033**
ICU *n* (%)				0.717
	Yes	2 (7.4%)	1 (1.6%)	
	No	25 (92.6%)	60 (98.4%)	
Hospital stay (days) median (IQR)		8 (7, 10)	7 (6, 7)	**<0.001**

**Table 2:** The 2018 FIGO staging system distribution.

FIGO Stage	Group A *n*(%) 27 (30.7%)	Group B *n*(%) 61 (69.3%)
IA	12 (44.4)	25 (41)
IB	0 (0)	5 (8.2)
IC	8 (29.6)	24 (39.3)
IIIA1	7 (26)	7 (11.5)

## Data Availability

In accordance with the journal’s guidelines, the data presented in this study are available upon request from the corresponding author for the reproducibility of this study if such is requested.
